# Regulation of the pentose phosphate pathway in cancer

**DOI:** 10.1007/s13238-014-0082-8

**Published:** 2014-07-12

**Authors:** Peng Jiang, Wenjing Du, Mian Wu

**Affiliations:** 1 School of Life Sciences, Tsinghua University, Beijing, 100084 China; 2 Hefei National Laboratory for Physical Sciences at Microscale, School of Life Sciences, University of Science and Technology of China, Hefei, Anhui, 230027 China; 3 Department of Cancer Biology, Perelman School of Medicine, Abramson Family Cancer Research Institute, University of Pennsylvania, Philadelphia, PA 19104 USA

**Keywords:** pentose phosphate pathway (PPP), G6PD, NADPH, glucose metabolism, cancer, cell proliferation

## Abstract

Energy metabolism is significantly reprogrammed in many human cancers, and these alterations confer many advantages to cancer cells, including the promotion of biosynthesis, ATP generation, detoxification and support of rapid proliferation. The pentose phosphate pathway (PPP) is a major pathway for glucose catabolism. The PPP directs glucose flux to its oxidative branch and produces a reduced form of nicotinamide adenine dinucleotide phosphate (NADPH), an essential reductant in anabolic processes. It has become clear that the PPP plays a critical role in regulating cancer cell growth by supplying cells with not only ribose-5-phosphate but also NADPH for detoxification of intracellular reactive oxygen species, reductive biosynthesis and ribose biogenesis. Thus, alteration of the PPP contributes directly to cell proliferation, survival and senescence. Furthermore, recent studies have shown that the PPP is regulated oncogenically and/or metabolically by numerous factors, including tumor suppressors, oncoproteins and intracellular metabolites. Dysregulation of PPP flux dramatically impacts cancer growth and survival. Therefore, a better understanding of how the PPP is reprogrammed and the mechanism underlying the balance between glycolysis and PPP flux in cancer will be valuable in developing therapeutic strategies targeting this pathway.

## Introduction

Tumor cells have long been known to reprogram their energy metabolism to meet high biogenetic demands in support of rapid and uncontrolled growth. For example, normal differentiated cells primarily employ mitochondrial oxidative phosphorylation to generate the energy and biomass required to support cellular processes; however, unlike normal tissues, most cancer cells display fundamental changes in nutrient metabolism and rely on aerobic glycolysis, a phenomenon called the Warburg effect (Vander Heiden et al., 2009; Warburg, 1956; Warburg et al., 1924). The enhancement of aerobic glycolysis provides cancer cells with a proliferative advantage by generating sufficient energy sources, such as adenosine 5′-triphosphate (ATP), and providing carbon intermediates for biosynthesis. In multicellular organisms, nutrient uptake and metabolism in most cells are tightly regulated by stringent control systems to prevent abnormal proliferation (Vander Heiden et al., 2009). However, cancer cells can overcome this metabolic surveillance by acquiring genetic mutations in genes such as key tumor suppressors and oncogenes. These genetic mutations can accumulate in cells over the course of an individual’s lifespan and functionally alter signaling pathways that sense or adjust aberrant metabolic programing. Aberrant changes in these signaling pathways increase the nutrient uptake and metabolism necessary to fuel cell survival and proliferation (DeBerardinis et al., 2008; Hsu and Sabatini, 2008). Nutrient metabolism, especially glucose metabolism, has been linked to growth control by both activation and genetic silencing of certain tumor genes, leading to uncontrolled cell proliferation, cell cycle arrest and even cellular aging (Cairns et al., 2011; Kroemer and Pouyssegur, 2008).

Recently, a glucose metabolic pathway, namely, the PPP, has been drawing significant attention for its newly uncovered role in the sensing of both intracellular and extracellular signals. Emerging evidence suggests that the PPP directly or indirectly provides reducing power to fuel the biosynthesis of lipids and nucleotides and sustains antioxidant responses to support cell survival and proliferation. Changing some cellular energy metabolic pathways, such as glycolytic flux, or genetic alteration of signaling pathways may substantially affect the PPP. In this review, we will discuss how the PPP is regulated and how the PPP and glycolysis, another important glucose metabolic pathway, are reciprocally regulated and balanced in the context of nutrient uptake and certain stress responses in cancer. We will also review the regulation of the PPP by several signaling pathways implicated in cell proliferation and discuss how this regulation can help cells meet the high demands of biogenesis during proliferation and detox ROS in persister survival.

## PPP, G6PD, glucose metabolism and biosynthesis

**Figure 1:**
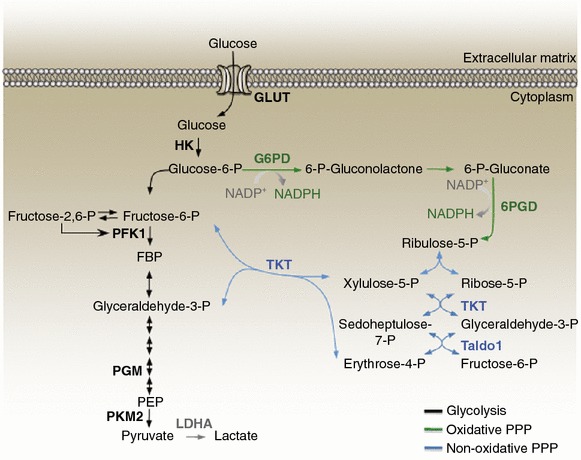
**A schematic representation of the PPP and glycolysis is shown**. The oxidative branch of the PPP yields NADPH that can be used in biosynthetic reactions for nucleotides, lipids and antioxidant defense. The reversible non-oxidative branch produces ribose-5-phosphate from oxidative branch as well as glycolytic intermediates. Solid black arrows represent glycolytic flux, green arrows represent the oxidative branch of the PPP, and light blue arrows represent multi-step processes of non-oxidative branch of PPP. For clarity, each component of the metabolic process has been abbreviated. PPP, pentose phosphate pathway; G6PD, glucose-6-phosphate dehydrogenase; 6PGD, 6-phosphogluconate dehydrogenase; TKT, transketolase; Taldo1, transaldolase; HK, hexokinase; GLUT, glucose transporters; PFK1, phosphofructokinse-1; PGM, phosphoglyceratemutase; PKM2, pyruvate kinase (PK)-M2; LDHA, lactate dehydrogenase A; FBP, fructose-1,6-bisphosphate; PEP, phosphoenolpyruvate

Glucose is a common fuel for multicellular organisms, entering cells through a glucose transporter (GLUT) and then being phosphorylated by hexokinase (HK) to form glucose-6-phosphate (G6P). G6P can be further metabolized by both the glycolytic pathway and the PPP; the latter is also known as the hexose monophosphate shunt (Fig. 1). G6P is then isomerized to fructose-6-phosphate (F6P) and metabolized through glycolysis to pyruvate by various glycolytic enzymes, leading to the production of ATP and other essential metabolites. In cancer cells, most pyruvate is converted to lactate instead of entering the mitochondria for further oxidation; this lactate is then released from the cells. G6P can also be dehydrogenated by G6PD in the oxidative branch of the PPP. This reaction is irreversible and rate-limiting under physiological conditions. The PPP includes an oxidative and non-oxidative branch. Whereas G6PD is the first and rate-limiting enzyme and acts as a control site in the oxidative branch, transketolase (TKT) and transaldolase are the two key enzymes in the non-oxidative branch (Kletzien et al., 1994; Stanton, 2012; Wood, 1986). In the oxidative phase, G6P is converted to 6-phosphoglucono-δ-lactone by G6PD. 6-phosphoglucono-δ-lactone is hydrolyzed to give rise to 6-phosphogluconate, which is then oxidatively decarboxylated by 6-phosphogluconate dehydrogenase (6PGD) to yield the five-carbon molecular ribulose 5-phosphate (Ru5P). During the oxidative phase, NADP^+^ acts as the electron acceptor in the two oxidative reactions, which are the first and the last reactions. Hence, one molecule of glucose oxidized and metabolized by the PPP can yield two molecules of NADPH. NADPH plays a vital role in both reductive biosynthesis and in the protection of cells from reactive oxygen species, which damage macromolecules and ultimately cause cell death. The other important molecule generated by the PPP is ribose-5-phosphate (R5P), which is an important precursor to many biomolecules, such as nucleotides. The five-carbon sugar R5P, which is generated from six-carbon glucose during the non-oxidative phase of the PPP, can be reversibly converted into the glycolytic intermediates glyceraldehyde 3-phosphate (G3P) and F6P by TKT and transaldolase. Therefore, glycolysis and gluconeogenesis coordinate with the PPP to control the cellular production of NADPH and R5P and to determine which phase of the PPP is activated. For example, if cells need more NADPH than R5P for antioxidant defense or reductive biosynthesis, such as for the production of fatty acids, R5P is converted into G3P and F6P, which are used to synthesize G6P by the gluconeogenic pathway. G6P is then channeled back into the PPP oxidative phase to produce more NADPH. In contrast, if cells need more R5P than NADPH to produce nucleotide precursors of DNA, G6P is converted into G3P and F6P by the glycolytic pathway, which is then converted into R5P by the non-oxidative phase of the PPP for nucleotide synthesis.

G6PD catalyzes the first irreversible reaction of the PPP and generates the reducing agent NADPH. Due to the crucial function of NADPH in scavenging cellular ROS, G6PD plays a critical role in antioxidant defense; indeed, G6PD-deficient cells are usually highly sensitive to oxidative stress. Because this enzyme is the rate-limiting enzyme in the PPP and acts as a “gatekeeper” of this pathway, the activity of G6PD directly reflects the flux of oxidative PPP and determines the flux partitioning between glycolysis and the PPP. Therefore, the expression and activity of this enzyme must be tightly controlled in cells. However, although many studies have reported that the activity of G6PD may be affected by numerous factors, the mechanism by which G6PD is regulated remains largely unknown. G6PD is present in both monomeric and dimeric forms in cells. Dimeric G6PD is the active form, but its monomeric form has little or no catalytic activity. G6PD exists in a state of monomer-dimer equilibrium in the cytosol under physiological conditions. Hypothetically, regulation of G6PD activity can be achieved by changing the monomer-dimer balance. Indeed, a recent study by us revealed that the tumor protein p53 controls G6PD activity by binding directly to G6PD and preventing its dimerization. Furthermore, we found that NADP^+^, which is known to activate G6PD by competing with NADPH for binding to this enzyme, plays a role in stabilizing G6PD dimers by blocking the p53-G6PD interaction (Jiang et al., 2011).

G6PD directly controls the PPP flux, which generates R5P for the biosynthesis of nucleotides and NADPH for reductive biosynthesis and ROS scavenging, and high G6PD activity is expected to result in an increase in the biosynthesis of lipids and DNA, which are both needed for cell division and proliferation. Thus, rapidly proliferating cells or cancer cells usually increase the PPP flux by activating G6PD to meet the bioenergetic demands during proliferation. Additionally, 6PGD, the other NADPH-generating enzyme of the oxidative pathway, may play a role in adjusting the flux of oxidative PPP. Similar to G6PD, 6PGD may also regulate cellular reductive biosynthesis and cell proliferation by modulating the production of NADPH and R5P.

## Glycolytic reprogramming and the Warburg effect

Cells ingest and metabolize glucose to generate G6P, which can be either metabolized by the PPP or the glycolytic pathway. Typically, cancer cells rely on glycolysis for energy production from glucose. In the 1920’s, the German physiologist Otto Warburg observed that cancer cells consume large amounts of glucose and convert it to lactate even in the presence of oxygen (called aerobic glycolysis or the Warburg effect) (Warburg, 1956; Warburg et al., 1924). Glucose is quickly metabolized through glycolysis and converted to 3-carbon lactate, which is excreted from cells instead of being further oxidized. Compared to normal cells, only two to four molecules of ATP can be generated from the metabolism of one molecule of glucose in cancer cells (Vander Heiden et al., 2009). However, because only ten reaction steps take place during aerobic glycolysis, cancer cells are able to produce ATP and accumulate glycolytic intermediates very quickly compared to cells utilizing mitochondrial oxidative phosphorylation, wherein glucose is completely catabolized. Thus, to some extent, aerobic glycolysis may give cancer cells an advantage in competing with normal tissues for nutrients. ATP was previously believed to be a critical and most likely limiting factor for cell proliferation. However, a growing body of evidence suggests that ATP, though important for the modulation and maintenance of cellular activities, is not limiting for cell growth (Lunt and Vander Heiden, 2011). Therefore, we have reason to believe that adopting aerobic glycolysis preferentially over oxidative phosphorylation, a phenomenon now known as the Warburg effect, is not detrimental to cancer cells. Rather, this bias may represent a profound advantage, i.e., the accumulation of intermediates (e.g., nucleotides, amino acids and lipids) that are helpful for biomass production.

Unlike other glucose metabolic pathways, glycolysis has been studied extensively, and its alteration by various signaling has been shown to influence cancer growth either directly or indirectly. Phosphoinositide 3-kinase (PI3K), which is well known as an oncoprotein, can regulate glucose consumption through the PI3K/AKT signaling pathway, thereby enhancing glucose transporter expression and phosphofructokinase activity (DeBerardinis et al., 2008). The phosphokinase 5′-AMP-activated protein kinase (AMPK) is another important modulator of metabolism that senses the cellular ratio of AMP to ATP and is activated by increases in this ratio. Furthermore, AMPK can be activated independently of the cellular AMP/ATP ratio by oxidative stress and other mechanisms (da Silva et al., 2006; Schulz et al., 2005; Stahmann et al., 2006; Sun et al., 2006; Towler and Hardie, 2007; Zmijewski et al., 2010; Zou et al., 2003). Upon activation, AMPK phosphorylates numerous substrates to regulate glucose uptake, mitochondrial biogenesis and gluconeogenesis suppression. Studies also reveal that AMPK activation leads to p53 phosphorylation and activation, resulting in the prevention of cell death under glucose deprivation conditions (Jones et al., 2005) and the promotion of replicative cell senescence (Jiang et al., 2013a). The oncogene Myc transcriptionally controls several metabolic genes via direct or indirect mechanisms (Dang, 2012; Gao et al., 2009; Wise et al., 2008). Interestingly, Myc-driven human cancer cells strongly depend on glutamine and are dramatically sensitive to glutamine starvation (Yuneva et al., 2007). Glutamine deprivation causes the rapid reduction of TCA cycle intermediates and cell apoptosis (Yuneva et al., 2007). Recent studies have also demonstrated that p53, the most frequently mutated human cancer gene, and its family member p73 are involved in the regulation of glucose and glutamine metabolism (Amelio et al., 2013; Berkers et al., 2013; Candi et al., 2014; Du et al., 2013; Fets and Anastasiou, 2013; Jiang et al., 2013c; Liang et al., 2013; Lunt and Vander Heiden, 2011; Shen et al., 2012). p53 enhances mitochondrial respiration through the activation of SCO2, which regulates the cytochrome c oxidase complex. The activation of SCO2 increases the efficiency of mitochondrial respiration (Matoba et al., 2006). The inhibition of aerobic glycolysis by p53 through the regulation of phosphoglyceratemutase 2 (PGM2), tumor protein 53-induced glycolysis and apoptosis regulator (TIGAR), glucose transporters 1 and 4 (GLUT1 and GLUT4), and glucose-6-phosphate dehydrogenase (G6PD), favors a reduction in glucose uptake and metabolism (Bensaad et al., 2006; Jiang et al., 2011; Kondoh et al., 2005; Schwartzenberg-Bar-Yoseph et al., 2004). Additionally, the regulation of phosphate-activated glutaminase (GLS2) reveals that p53 may have a role in modulating glutamine metabolism (Hu et al., 2010; Suzuki et al., 2010), and the mutual regulation of malic enzymes (MEs) and p53 demonstrates that p53 rigorously regulates and couples glutamine metabolism to cell fate decisions. Indeed, p53 deficiency results in defective cell growth arrest (senescence) and an increase in proliferation (Jiang et al., 2013a). Unlike p53, the evidence for p73 in energy metabolism was a complete mystery until its role in the transcriptional regulation of G6PD and cytochrome c oxidase subunit 4 isoform 1 (Cox4i1) was recently uncovered (Du et al., 2013; Rufini et al., 2012). All these findings suggest that the dysregulation of important oncogenes or tumor suppressors may significantly impact cellular regulation systems that function in adjusting metabolism.

Two primary nutrients captured and utilized by cancer cells are glucose and glutamine. These two nutrients contribute to most of the cellular carbon sources used for biogenesis (Lunt and Vander Heiden, 2011). Glucose catabolism generates ATP, NADPH and other biomasses for reductant biosynthesis and ROS detoxification. In association with the TCA cycle, glutamine metabolism provides not only a carbon source but also NADH, NH_3_^+^ and other essential intermediates for lipid biosynthesis, amino acid synthesis (Lunt and Vander Heiden, 2011), and cellular acid detoxification (Huang et al., 2013). Unlike glutamine, which is only required for some human cancers, glucose seems to universally be the most critical nutrient for the growth and proliferation of both normal and cancer cells. Human head and neck squamous carcinoma cells (HNSCCs) are highly dependent on glucose. This dependence is particularly true of cells that harbor a p53 deletion or loss of function, and glycolytic inhibition has profound global metabolic consequences in these cells. Thus, cells cultured in glucose-free medium will quickly die or enter cell cycle arrest (Sandulache et al., 2011). However, the enhanced flux of glucose to lactate is insufficient to promote cell replication (Hsu and Sabatini, 2008). Cells are largely composed of proteins and ribonucleic acids and must also address waste and neutralize superoxide and hydrogen peroxide, which accompany accelerated nutrient metabolism. Therefore, other metabolic pathways must also be reprogrammed in cancer cells to provide the building blocks for cell replication (Koppenol et al., 2011).

## Oncogenic regulation of the PPP and the Warburg effect

The PPP is a major glucose catabolic pathway that links glucose metabolism to the biosynthesis of the nucleotide precursor ribose and to NADPH production. This latter process is essential for both antioxidant defense and reductive biosynthesis, such as lipid synthesis. Historically, however, much less attention has been paid to the importance of the PPP in cancer growth, and alterations to the PPP in cancer cells were not well understood. Recent studies have demonstrated that the PPP, together with glycolysis, coordinates glucose flux and supports the cellular biogenesis of macromolecules and energy production. Glycolysis provides cells with energy for biogenesis; however, large amounts of lipid and nucleotide precursors are needed to sustainably support the uncontrolled proliferation of cancerous cells. Lipids are used in the construction of cell membranes and as energy storage, and nucleotides serve as substrates for continuous DNA replication. To meet these biosynthetic demands, cancer cells are metabolically reprogrammed to direct glucose flux into the PPP. Indeed, growing evidence suggests that, similar to glycolysis, higher PPP flux is present in many human cancers. Further studies have also revealed that alterations of the PPP significantly contribute to tumor growth and survival under certain stress conditions. For example, during oxidative stress, cancer cells will shut down the glycolytic pathway and thus increase glucose flux through the PPP to produce more NADPH for antioxidant defense. The evidence that PPP flux is fundamentally higher in some human cancer cells supports the idea that the PPP may play a critical role in meeting the bioenergetic demands of cancer cell proliferation and contribute to the Warburg effect.

**Figure 2:**
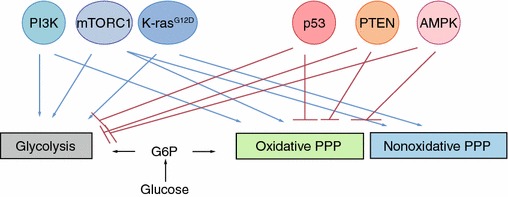
**Regulation ofsome key oncoproteins and tumor suppressors on glycolysis and PPP**. Tumor proteins, including PI3K and K-ras^G12D^ that are often activated in cancers, positively regulate glycolysis and PPP. Consistently, inactivation of tumor suppressors such as p53, PTEN or AMPK leads to enhancement of both glycolysis and PPP flux and supports cell proliferation. PI3K, phosphoinositide 3-kinase; AMPK, AMP-activated protein kinase; PTEN, phosphatase and tensin homologue; mTORC1, mTOR complex 1

As mentioned above, the PPP and glycolysis are coordinately regulated to support cell growth and survival. In cancer cells, the activation of glycolysis may also be accompanied by an increase in PPP activity for biosynthesis. Cancer cells often bypass growth checkpoints through genetic mutations in essential genes (Fig. 2). One of the most frequently mutated genes is p53, and p53 mutations or loss of function lead to the enhancement of both glycolytic and PPP flux (Bensaad et al., 2006; Jiang et al., 2011). p53 deficiency reduces the expression of TIGAR, which has a role in suppressing glycolysis by lowering intracellular levels of fructose-2,6-bisphosphate (F-2,6-P_2_). F-2,6-P_2_ is a strong allosteric activator of phosphofructokinse-1 (PFK1), and the reduction of F-2,6-P_2_ results in decreased PFK1 activity and glycolytic flux (Bensaad et al., 2006). p53 suppresses the PPP by directly binding to G6PD and repressing its enzyme activity. However, the ability to inhibit G6PD is restricted to wild type p53. A cancer-associated mutation of p53 has been shown to lose the ability to block G6PD activity (Jiang et al., 2011). Therefore, it can be hypothesized that in cancer cells, p53 mutations may liberate G6PD and activate PFK1, causing increased PPP flux and glycolysis. These findings demonstrate the central role of p53 in the tight control of intracellular glucose metabolism. Another interesting finding is that the oncogene K-ras^G12D^ stimulates glycolysis and drives glycolysis intermediates into the nonoxidative PPP in pancreatic ductal adenocarcinoma (PDAC) (Ying et al., 2012), which commonly harbors p53 mutations (Hezel et al., 2006). K-ras^G12D^ inactivation leads to decreases in glycolytic enzyme expression, glycolysis, reducing nonoxidative PPP and ribose biogenesis.

PI3K, a well-known oncoprotein, regulates glucose consumption through the PI3K/AKT signaling pathway to enhance glucose transporter expression and phosphofructokinase activity, thereby promoting glycolysis (DeBerardinis et al., 2008). PI3K activation initiates a signal transduction cascade that stimulates cell growth and survival, and the activation of the serine-threonine kinase AKT by PI3K is widely implicated in cancers (Engelman, 2009). The activation of AKT by PDK1 (Manning and Cantley, 2007) and mammalian target of rapamycin complex 2 (mTORC2) (Zoncu et al., 2011) can phosphorylate a series of cellular proteins, such as glycogen synthase kinase 3α (GSK3α), GSK3β, forkhead box O transcription factors (FoxO), MDM2, and BCL2-associated agonist of cell death (BAD), leading to cell survival and cell cycle entry (Bader et al., 2005; Cantley, 2002; Engelman, 2009; Engelman et al., 2006; Shaw and Cantley, 2006). In addition to its regulatory role in glycolysis, a study published several years ago indicated that the PI3K/AKT signaling pathway may regulate G6PD in a transcription-dependent manner, although the mechanism behind this process remains uncharacterized (Wagle et al., 1998). The phosphatase and tensin homologue (PTEN), a p53 target gene, is also a tumor suppressor that is frequently mutated or deleted in human cancers (Bonneau and Longy, 2000; Cantley and Neel, 1999; Simpson and Parsons, 2001). PTEN has unique PtdIns(3,4,5)P3 lipid phosphatase activity that negatively regulates the PI3K signaling pathway by dephosphorylating phosphatidylinositol-3,4,5-trisphosphate (PIP3) (Maehama and Dixon, 1998). Consistent with its role in cell proliferation and tumorigenesis, a recent study found that PTEN elevation results in reduced glucose and glutamine uptake and increased mitochondrial oxidative phosphorylation through PI3K-dependent and -independent pathways (Garcia-Cao et al., 2012). Intriguingly, similar to p53, PTEN appears to have a role in suppressing G6PD and its enzymatic activity (Hong et al., 2013). AMPK, which plays a central role in the regulation of cellular energy homeostasis, was also shown to negatively regulate aerobic glycolysis (Faubert et al., 2013) and G6PD expression in cancer cells (Kohan et al., 2009; Stanton, 2012; Vander Heiden et al., 2009). Moreover, activation of the metabolic regulatory network downstream of mTOR complex 1 (mTORC1) has been demonstrated to cause reprogramming of the essential metabolic pathways, including glycolysis, glutaminolysis and the PPP. mTORC1 activation increases the levels of glycolysis and PPP metabolites through induction of the expression of glycolytic genes and G6PD. Of note, the induction of G6PD by mTORC1 is dependent on the SREBP (sterol regulatory element-binding protein) transcription factors. The knockdown of SREBP1 reduces G6PD expression in Tsc2-deficient cells, whereas the overexpression of the processed form of SREBP1 increases G6PD expression in HEK293 cells (Duvel et al., 2010). Collectively, these findings suggest that in cancer cells, oncogenic mutations usually increase both glycolysis and PPP flux to support the biogenesis required for rapid growth and division. However, this companion relationship between PPP and glycolysis in cancers does not exist when cells are exposed to certain stresses, such as oxidative stress or DNA damage. To prevent such stress-induced death, cells usually stimulate only one of the pathways if the other is blocked or attenuated by stress (see detailed discussion below).

## Metabolic alterations in the oxidative stress-mediated PPP

**Figure 3:**
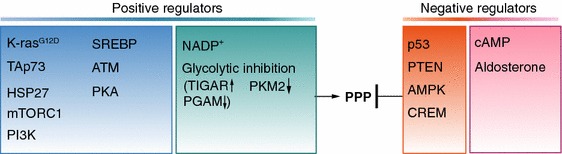
**PPP is either positively or negatively regulated by numerous factors as indicated**. ATM, the ataxia-telangiectasia mutated kinase; cAMP, cyclic adenosine monophosphate; CREM, cyclic AMP-response element modulator; PKA, protein kinase A

The central role of the PPP in tumor metabolism has attracted more attention in recent years. Emerging evidence suggests that the PPP is tightly and meticulously controlled in cells and that its abnormal regulation leads to uncontrolled biosynthesis. As the first and rate-limiting enzyme in the PPP, G6PD has recently received significant attention. Its enzymatic activity is mediated by various signals, and it acts as a sensor of cellular NADP^+^ levels (Fig. 3). Increased NADP^+^ activates G6PD by competing with NADPH for binding to this enzyme. Therefore, alteration of the cellular NADP^+^/NADPH ratio by oxidative stress or other metabolic reprogramming can be expected to impact the PPP flux through G6PD. For example, the stimulation of cellular ROS generation caused by either oxidative stress or inhibition of NADPH-generating pathways would lead to increases in the cellular NADP^+^/NADPH ratio, and the increased NADP^+^ in turn would activate G6PD and the PPP to produce NADPH to compensate for the stress. During cell proliferation, NADPH is required for reductive biosynthesis and nucleotide synthesis, and the sustained consumption of NADPH will be balanced by increased NADP^+^ to achieve and maintain an optimal physiological NADP^+^/NADPH ratio for biosynthesis.

The major NADPH-producing enzymes include G6PD, malic enzymes (MEs) and isocitrate dehydrogenases (IDHs). As PPP is a primary source of cytosolic NADPH, G6PD (alone or together with 6PGD) is expected to make a large contribution to the production of NADPH. MEs and IDHs are associated with the TCA cycle, and are presumably important for the metabolism of glutamine. Our recent study demonstrated for the first time that MEs significantly contribute to cellular NADPH generation and are vital for tumor growth (Jiang et al., 2013a). The cytosolic ME1 and mitochondrial ME2 catalyze the oxidative decarboxylation of malate to yield pyruvate, CO_2_, and NAD(P)H. Both ME1 and ME2 strongly influence cellular NADPH and lipid production, but intriguingly, whether ME1 or ME2 plays a more dominant role in these processes may be cell-type dependent. For example, in certain types of human cancer cells and in primary fibroblast IMR90 cells, ME2 more profoundly affects NADPH production and lipogenesis, whereas ME1 has a much smaller effect. IDHs catalyze the reversible oxidative decarboxylation of isocitrate to α-ketoglutarate (α-KG) while reducing NADP^+^ to NADPH. Inhibition of IDH activities has been found to be associated with decreased NADPH production in both normal human brain and glioma cells (Bleeker et al., 2010). However, IDH activity is still believed to contribute relatively little to NADPH production in general. Additionally, recent studies uncovered somatic mutations in the two IDH isoforms, IDH1 and IDH2, which occur at high frequencies in cancers, including gliomas, astrocytomas, chondromas and acute myeloid leukemia (AML) (Parsons et al., 2008; Ward et al., 2012; Ward et al., 2010; Yan et al., 2009; Zhao et al., 2009). In contrast to wild type IDHs, mutant IDHs are associated with a decrease rather than an increase in NADPH. A possible explanation for this observation is that mutant IDH1 and IDH2 proteins not only lose the ability to catalyze the isocitrate–α-KG reaction but also convert α-KG to R(-)-2-hydroxyglutarate ([R]-2HG) by expending NADPH (Dang et al., 2010; Ward et al., 2012; Ward et al., 2010). Again, given the important role of NADPH in maintaining cellular functions and its activity as a cofactor for these enzymes, breaking the balance between NADP^+^ and NADPH by blocking one or two of these pathways would influence the activity of the others. Therefore, a compensatory effect between these NADPH-generating enzymes is expected (and, indeed, has been confirmed by our unpublished findings).

**Figure 4:**
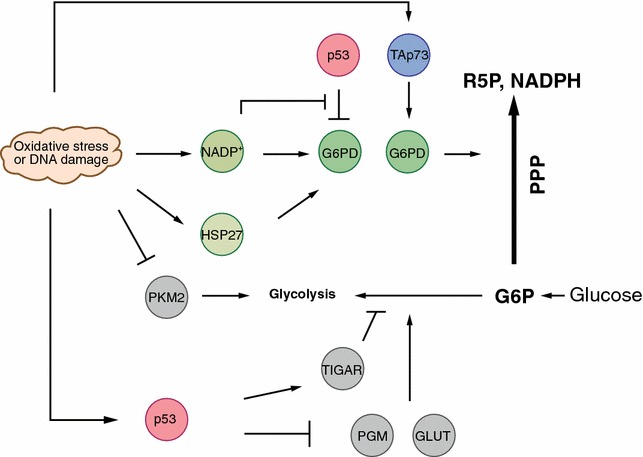
**Metabolic alterations of PPP flux in response to oxidative stress and genotoxic stress**. Oxidative stresses reprogram PPP by oncogenic regulation through activation of TAp73 or HSP27, or by metabolic alteration of glycolysis to build up the glycolytic intermediates

As mentioned above, the glycolytic pathway and the PPP are two metabolic pathways that are tightly connected and cooperatively adjust glucose uptake and metabolism. Several studies have revealed that blocking one of these two pathways would have a dramatic impact on the other under certain stress conditions. For example, the inhibition of G6PD in human colon cancer HCT116 cells or MEF cells leads to increased glycolytic flux and lactate production within a relatively short period of time (Jiang et al., 2011). As expected, the long-term knockdown of G6PD will result in cell growth arrest and even cell senescence. In contrast, to increase NADPH production, cells can acquire the ability to increase PPP flux by suppressing glycolysis and redirecting glycolytic intermediates into the PPP (Fig. 3). However, the exact pathophysiology remains unclear, and different underlying mechanisms have been proposed, including protein expression and modification as well as protein-protein interactions. One example is the upregulation of TIGAR. TIGAR overexpression causes the inhibition of glycolysis and G6P accumulation, which in turn stimulates the PPP to promote NADPH production and cell growth (Bensaad et al., 2006; Cheung et al., 2013). Furthermore, Pyruvate kinase (PK)-M2 is an isoform of PK that catalyzes the conversion of phosphoenol pyruvate to pyruvate. Conditions with high ROS levels reduce the activity of PK-M2 through the oxidation of Cys(358), leading to a build-up of glycolytic intermediates to be shunted to the PPP (Anastasiou et al., 2011). More recently, TAp73, the transcriptionally competent isoform of the p53 family protein p73, was identified as a transcriptional regulator of G6PD (Du et al., 2013; Jiang et al., 2013b, c). The ataxia-telangiectasia mutated kinase (ATM), which regulates DNA damage responses, stimulates the activity of G6PD through the phosphorylation of HSP27, which is an activator of G6PD (Cosentino et al., 2011; Jiang et al., 2013c). Because numerous signaling events can regulate PPP activity, indicating that the PPP is critical for cell survival and proliferation, the precise regulation of this pathway would appear to be particularly important with regard to cancer cells. Indeed, most studies have demonstrated that the PPP is coordinately reprogrammed in response to both oxidative and genomic damage stresses (Fig. 4). When cells are exposed to oxidative stress, many responses are induced, including increased levels of cellular NADP^+^ and ROS. Oxidative stress can induce p53 activation, which promotes TIGAR expression and suppresses the expression of PGM and GLUT, leading to the inhibition of glycolysis. Moreover, ROS inhibits PKM2 and thus represses glycolysis. The accumulation of glycolytic intermediates caused by glycolytic inhibition channels glucose flux into the PPP. Meanwhile, the inhibition of G6PD by the activation of p53 is blocked by the increased NADP^+^, opening the door for PPP activation. Of note, in the absence of such stress, p53 inactivation enhances PPP flux (Jiang et al., 2011), demonstrating that the p53-mediated inhibition of G6PD likely overrides the effects of TIGAR and other regulators. The different mechanisms for the regulation of the PPP and glycolysis by p53 may enable p53 to differentially adjust the PPP and glucose metabolism in cells (Jiang et al., 2013c). Oxidative stress and genomic damage can also activate TAp73 and ATM/HSP27 signaling pathways to directly stimulate G6PD. If the G6PD-regulated PPP is activated, more NADPH will be available to reduce ROS and protect cells from DNA damage.

In addition, there are some small molecules that can affect PPP activity and contribute to ROS generation (Fig. 3). Adrenaline and cyclic adenosine monophosphate (cAMP) are able to inhibit G6PD activity through PKA-mediated phosphorylation *in vitro*, which might contribute to oxidative stress *in vivo* (Costa Rosa et al., 1995; Xu et al., 2005; Zhang et al., 2000). Furthermore, aldosterone decreases both the expression and activity of endothelial G6PD through the cyclic AMP-response element modulator (CREM) to inhibit CREB-mediated G6PD transcription, resulting in increased oxidative stress (Leopold et al., 2007).

## Future perspective and clinical potential

G6PD is important for growth and development, and severe G6PD deficiency is lethal to embryos (Longo et al., 2002). One of the likely explanations is the key role of G6PD in the production of NADPH for the detoxification of ROS. Nevertheless, given the importance of NAPDH for the balance between reductive biosynthesis and redox and the importance of R5P for nucleotide synthesis, the inhibition of the PPP might be an attractive way to target rapidly growing tumor cells (Jones and Schulze, 2012). As discussed above, increased PPP flux or G6PD activity has been shown to be frequently found in some cancer cell lines and to be correlated with some of the key oncogenic mutations in human cancers. Consistent with this observation, G6PD is overexpressed in many human cancers (Jiang et al., 2013b), including diffuse large B-cell lymphoma (Compagno et al., 2009; Rosenwald et al., 2002), uterine corpus leiomyosarcoma (Quade et al., 2004) and lung adenocarcinoma (Stearman et al., 2005; Su et al., 2007). More evidence has been provided by a study showing that G6PD plays a crucial role in promoting malignant cell survival and cell proliferation (reviewed by Stefania Filosa and her colleagues (Manganelli et al., 2013)). An interesting finding is that the ectopic expression of G6PD in NIH3T3 cells (a mouse embryonic fibroblast cell line) promotes anchorage-independent cell growth and increases the intracellular levels of NADPH and glutathione (Kuo et al., 2000).

Furthermore, we and other groups have confirmed that G6PD plays an important role in supporting cancer cell growth (Du et al., 2013; Jiang et al., 2011; Jiang et al., 2013b; Tian et al., 1998). In particular, we have elucidated the mechanisms for the regulation of the PPP and explained how cancer cells direct glycolytic intermediates into biosynthesis. The tumor suppressor p53 inhibits the PPP through direct inactivation of G6PD (Jiang et al., 2011). p53 binds to G6PD and inhibits G6PD dimerization, leading to decreases in G6PD activity, glucose consumption, NADPH production and biosynthesis. Interestingly, tumor-associated p53 mutants fail to inhibit G6PD. Therefore, in p53 mutant cancers, the enhanced PPP glucose flux may drive glucose consumption and direct glucose flux towards biosynthesis. TAp73, a p53 family member, has been shown to increase the expression of G6PD by binding to the G6PD gene and enhancing its transcription (Du et al., 2013). TAp73, unlike p53, is rarely mutated but is frequently highly expressed in human tumor cells. TAp73 is able to increase glucose uptake, NADPH production and nucleotide biosynthesis through the upregulation of G6PD. Interestingly, G6PD reintroduction in some types of human cancer cells significantly rescues the growth of TAp73-deficient cells and almost completely restores the proliferation of human lung cancer H1299 cells expressing p73 siRNA (small interfering RNA) (Du et al., 2013; Jiang et al., 2013b). These findings suggest that G6PD may act as an oncogene and could become another attractive potential target for anti-cancer drug development. Additionally, a combination of oxythiamine, a general TKT inhibitor, and the G6PD inhibitor DHEA has been shown to magnify the inhibition of cancer cell growth (Langbein et al., 2008). 2-Deoxy-D-glucose (2DG), a glucose analog, and 6-aminonicotinamide (6AN), an inhibitor of 6PGD, have also been reported to enhance radiosensitivity in human gliomas and squamous carcinoma cell lines (Manganelli et al., 2013; Varshney et al., 2005). Therefore, a combination of targeting the PPP and other metabolic pathways may be an effective approach to selectively suppress cancer cell growth. Moreover, many anticancer chemotherapies, such as 5-fluorouracil (5-FU) and adriamycin, act as mutagens to induce genetic damage and produce ROS. Therefore, the inhibition of PPP or G6PD may enhance the sensitivity of cancer cells to these chemotherapeutic agents (Jones and Schulze, 2012). Although many more clinical studies are needed, targeting G6PD or the PPP may be an exciting potential approach for anti-cancer therapy. Furthermore, more than 400 million people worldwide have been estimated to be G6PD deficient. G6PD deficiency causes certain disorders, such as mild anemia, but no other serious health issues. Thus, studying whether G6PD deficiency confers an advantage against the development of cancer would also be interesting. Collectively, gaining a better understanding of how the PPP is regulated and what selective advantage(s) this alteration provides for cancer cells may have therapeutic value in the fight against cancer.

## Abbreviations

6PGD: 6-phosphogluconate dehydrogenase
AMPK: AMP-activated protein kinase
ATM: the ataxia-telangiectasia mutated kinase
cAMP: cyclic adenosine monophosphate
CREM: cyclic AMP-response element modulator
FBP: fructose-1,6-bisphosphate
G6PD: glucose-6-phosphate dehydrogenase
GLUT: glucose transporters
HK: hexokinase
LDHA: lactate dehydrogenase A
mTORC1: mTOR complex 1
PEP: phosphoenolpyruvate
PFK1: phosphofructokinse-1
PGM: phosphoglyceratemutase
PI3K: phosphoinositide 3-kinase
PKA: protein kinase A
PKM2: pyruvate kinase (PK)-M2
PPP: pentose phosphate pathway
PTEN: phosphatase and tensin homologue
TKT: transketolase
